# Leveraging Transfer Learning and Attention Mechanisms for a Computed Tomography Lung Cancer Classification Model

**DOI:** 10.7759/cureus.87071

**Published:** 2025-06-30

**Authors:** Kian A Huang, Vishnu Venkitasubramony, Neelesh S Prakash

**Affiliations:** 1 Radiology, University of South Florida Morsani College of Medicine, Tampa, USA

**Keywords:** artificial intelligence, convolutional neural networks, ct imaging, deep learning, lung cancer, medical image classification, resnet50v2, squeeze-and-excitation

## Abstract

Background

Lung cancer is the leading cause of cancer-related mortality worldwide, with late-stage diagnosis contributing to poor survival rates. Early detection remains a critical challenge, hindered by diagnostic delays, radiologist shortages, and the limitations of current imaging workflows. Recent advances in artificial intelligence (AI), particularly deep learning, offer new avenues to enhance diagnostic accuracy and efficiency in radiology.

Objective

To develop and evaluate a deep learning model integrating Residual Network 50 Version 2 (ResNet50V2) with Squeeze-and-Excitation (SE) blocks for automated classification of lung cancer subtypes from computed tomography (CT) images.

Methods

A total of 1,000 anonymized lung CT images were obtained from a publicly available Kaggle dataset, categorized into four classes: adenocarcinoma, large cell carcinoma, squamous cell carcinoma, and normal tissue. The dataset was split into training (70%), validation (10%), and test (20%) sets. A fine-tuned ResNet50V2 architecture with SE blocks was used to enhance channel-wise feature recalibration. The model was trained using categorical cross-entropy loss with label smoothing and optimized via Adam. Performance was evaluated using accuracy, area under the receiver operating characteristic curve (AUC), precision, recall, and F1-score.

Results

The model achieved a test accuracy of 90.16% and an overall AUC of 0.9815. Class-wise AUCs were high across all categories: 0.9523 for adenocarcinoma, 0.9879 for large cell carcinoma, 0.9977 for normal tissue, and 0.9880 for squamous cell carcinoma. Precision ranged from 0.81 (large cell carcinoma) to 1.00 (normal tissue), while recall ranged from 0.85 (adenocarcinoma) to 0.98 (large cell carcinoma). F1-scores were consistently strong, ranging from 0.88 to 0.96.

Conclusion

The integration of SE blocks with ResNet50V2 yielded a high-performing model capable of accurately classifying lung cancer subtypes from CT images. The approach shows promise for assisting radiologists in diagnostic decision-making, particularly in settings with limited expert availability. Future work should focus on external validation, model interpretability, and exploration of emerging architectures such as Vision Transformers for enhanced performance and clinical adoption.

## Introduction

Lung cancer remains the leading cause of cancer-related deaths globally, accounting for approximately 1.8 million deaths annually, with a particularly high burden in both high-income and low-resource settings [1]. In the United States alone, lung and bronchus cancer ranks as the second most common cancer in both men and women, with over 226,500 new cases and an estimated 124,730 deaths expected in 2025 [2]. Despite advances in treatment and management, the five-year survival rate for lung cancer remains low, primarily due to late-stage diagnosis [1,2].

Early detection is crucial to improving outcomes, yet current diagnostic pathways are often hindered by significant limitations. Lung cancer is frequently detected only at advanced stages, when curative interventions are less effective [3]. Diagnostic bottlenecks stem from delays in imaging interpretation, limited accessibility to skilled radiologists, and invasive procedures such as biopsies, which carry risk and require significant clinical resources [4]. Additionally, radiologist burnout and cognitive overload in high-volume healthcare environments further impair the speed and accuracy of image-based diagnosis [4].

Artificial intelligence (AI), and more specifically deep learning, has emerged as a promising tool to support clinicians in overcoming these challenges [4,5]. AI refers to the simulation of human intelligence processes by machines, particularly computer systems capable of learning and problem-solving [4]. Deep learning, a subfield of AI, leverages layered neural networks, modeled loosely on the human brain, to automatically learn complex features from large datasets [5]. In medical imaging, deep learning models, especially convolutional neural networks (CNNs), have demonstrated superior performance in tasks such as tumor detection, organ segmentation, and disease classification across modalities including radiography, CT, MRI, and histopathology [4,5].

One notable CNN architecture is residual network 50 version 2 (ResNet50V2), a 50-layer residual network that improves gradient flow through skip connections [6]. This design enables deeper networks to be trained efficiently without vanishing gradients, making ResNet50V2 especially suitable for fine-grained visual classification tasks such as cancer subtype identification [6]. To further enhance feature representation, attention mechanisms have been integrated into CNNs, allowing models to selectively focus on the most informative regions of an image. Among these mechanisms, the Squeeze-and-Excitation (SE) block is particularly effective. SE blocks recalibrate channel-wise feature responses by explicitly modeling interdependencies between channels, thereby enhancing the discriminative power of the network [7]. This is achieved through a two-step process: a "squeeze" operation that condenses global spatial information into a channel descriptor, followed by an "excitation" step that adaptively recalibrates channel-wise feature responses [6]. Previous literature has shown SE to be a superior attention mechanism to use in ResNet50V2 models in MRI brain image classification compared to other mainstream choices [8].

Building on this foundation, we propose a deep learning model that integrates a fine-tuned ResNet50V2 architecture with SE blocks to classify lung cancer subtypes from CT lung images. By combining the residual learning capacity of ResNet with the channel attention mechanism of SE, our model aims to improve accuracy in distinguishing between adenocarcinoma, large cell carcinoma, squamous cell carcinoma, and normal lung tissues, offering a promising step toward AI-assisted diagnostic radiology in clinical practice.

## Materials and methods

This study utilized a publicly available dataset of anonymized lung CT scan images (n = 1000) hosted on Kaggle, pre-allocated and pre-randomized into three groups: a training set (70%), a testing set (20%), and a validation set (10%). The dataset was organized into four diagnostic categories: adenocarcinoma, large cell carcinoma, squamous cell carcinoma, and normal lung tissue. Each image was pre-labeled by expert annotation, and the dataset was manually reviewed to confirm labeling integrity. Due to natural prevalence variation among lung cancer subtypes, the dataset was imbalanced, with adenocarcinoma and squamous cell carcinoma comprising the majority of the samples. To mitigate the impact of this imbalance, class weights were computed using the class_weight utility and applied during training, which increases the sensitivity of classes with fewer data points.

The backbone architecture consisted of ResNet50V2, a 50-layer CNN pre-trained on ImageNet, selected for its ability to capture deep spatial hierarchies via residual connections. Fine-tuning was initiated from the conv3_block1_out layer, preserving early-layer feature extraction while enabling domain-specific adaptation in higher layers. To enhance channel-wise feature recalibration, SE blocks were integrated into the architecture, allowing dynamic weighting of features based on global context. The network concluded with a global average pooling layer followed by two dense layers of 1024 and 512 neurons, respectively, each with Rectified Linear Unit (ReLU) activation. A dropout layer (rate = 0.5) was placed between them to reduce overfitting. The final layer used softmax activation to yield normalized probabilities across the four classes.

Model training was conducted using the Adam optimizer with an initial learning rate of 1e-4. The loss function was categorical cross-entropy with label smoothing (set to 0.1) to regularize the output distribution and reduce overconfidence. Input CT images were resized to 224 × 224 pixels and rescaled to the zero-to-one intensity range. Three training callbacks were employed: EarlyStopping halted training when validation loss plateaued for 10 epochs; ReduceLROnPlateau decreased the learning rate by a factor of 0.5 after three stagnant validation epochs; and ModelCheckpoint preserved the weights of the best-performing model based on validation accuracy. The batch size was set to 16, and training was conducted for up to 100 epochs, though EarlyStopping typically concluded the training earlier. All experiments were conducted in TensorFlow 2.12 using the Keras backend.

## Results

**Figure 1:**
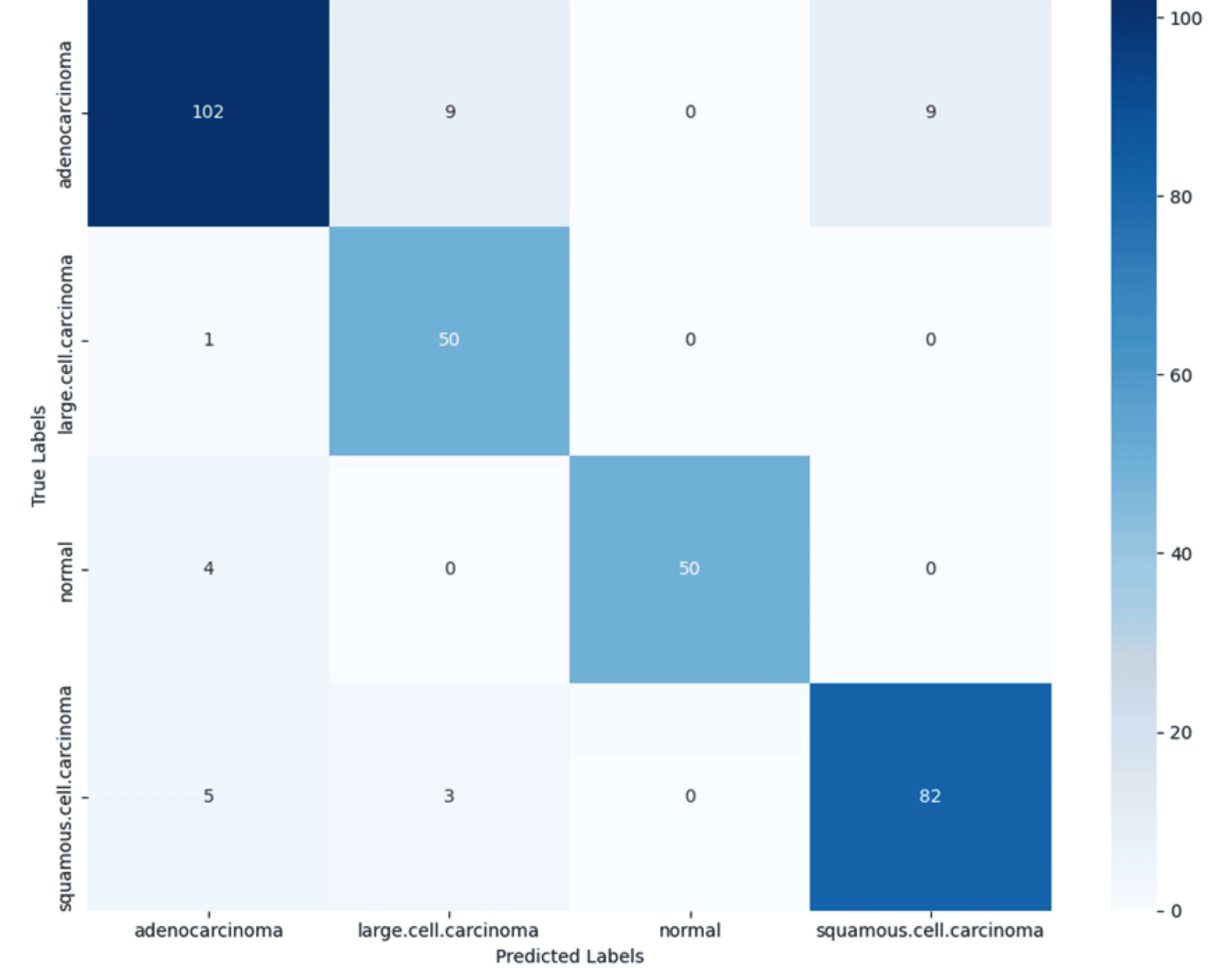
Confusion Matrix of Lung Cancer Classification The confusion matrix illustrates the performance of the proposed model in classifying four histological categories: adenocarcinoma, large cell carcinoma, squamous cell carcinoma, and normal tissue. Each cell represents the number of samples classified into a specific category, with the diagonal showing correct classifications.

Model performance was quantitatively assessed using accuracy and area under the receiver operating characteristic curve (AUC). Accuracy measures the proportion of correctly classified instances among all predictions. The proposed model achieved a validation accuracy of 91.67% and a test accuracy of 90.16%, indicating consistent generalization from training to unseen data. AUC, a robust metric for evaluating classification models, particularly in imbalanced datasets, quantifies the model's ability to distinguish between classes. An AUC of 1.0 represents perfect classification, while an AUC of 0.5 indicates no discriminative power. The overall AUC achieved by the model was 0.9815, reflecting high discriminative ability. As shown in Table 1, class-wise AUCs were also high: 0.9523 for adenocarcinoma, 0.9879 for large cell carcinoma, 0.9977 for normal tissue, and 0.9880 for squamous cell carcinoma, suggesting excellent model performance across all lung tissue categories. In addition to accuracy and AUC, model performance was further characterized using precision, recall, and F1-score, which provide a more detailed view of class-wise prediction quality. Precision, defined as the proportion of true positive predictions among all positive predictions made by the model, was highest for normal tissue (1.00), indicating perfect prediction without false positives. Precision for adenocarcinoma and squamous cell carcinoma were both strong, at 0.91 and 0.90, respectively, while large cell carcinoma had a lower precision of 0.81, suggesting a greater incidence of false positives in that class. Recall, which quantifies the ability of the model to identify all actual positive instances (i.e., true positives divided by the sum of true positives and false negatives), was highest for large cell carcinoma at 0.98, demonstrating the model's exceptional sensitivity for this class. Normal tissue and squamous cell carcinoma also showed high recall values of 0.93 and 0.91, respectively. Adenocarcinoma had a slightly lower recall of 0.85, indicating some missed detections. F1-score, the harmonic mean of precision and recall, captures the balance between these two metrics. The F1-scores were uniformly strong across classes: 0.96 for normal tissue, 0.91 for squamous cell carcinoma, and 0.88 for both adenocarcinoma and large cell carcinoma. These values reflect the model’s balanced ability to minimize both false positives and false negatives in each category. A confusion matrix provides a detailed breakdown of classification performance by comparing predicted versus actual labels. As illustrated in Figure 1, the matrix for this model displayed strong diagonal dominance, with the majority of predictions correctly aligned with true labels. 

**Table 1:** Performance Metrics for Lung Cancer Classification This table summarizes the classification performance of the proposed deep learning model across four histological categories: adenocarcinoma, large cell carcinoma, squamous cell carcinoma, and normal tissue. Metrics reported include precision, recall, F1-score, area under the curve (AUC), and the number of samples in each class.

Class	Precision	Recall	F1-Score	Sample Size	AUC
Adenocarcinoma	0.91	0.85	0.88	120	0.9523
Large Cell Carcinoma	0.81	0.98	0.88	51	0.9879
Normal Tissue	1.00	0.93	0.96	54	0.9977
Squamous Cell Carcinoma	0.90	0.91	0.91	90	0.9880

## Discussion

The results of this study demonstrate that a deep learning model combining a fine-tuned ResNet50V2 architecture with SE blocks can achieve high accuracy and discriminative power in classifying lung cancer subtypes from CT images. With a test accuracy of 90.16% and an overall AUC of 0.9815, the model exhibits robust performance across diverse classes, including adenocarcinoma, large cell carcinoma, squamous cell carcinoma, and normal lung tissue. These findings contribute meaningfully to the growing body of research supporting the application of deep CNNs in medical image classification and reinforce the utility of attention-enhanced architectures in improving diagnostic performance [5-7].

The high per-class AUCs, particularly 0.9977 for normal tissue and 0.9880 for squamous cell carcinoma, underscore the model’s capacity to distinguish both malignant and benign phenotypes, a critical requirement for real-world clinical application. The perfect precision for normal lung tissue (1.00) indicates that the model makes no false-positive predictions in this category, thereby reducing unnecessary clinical interventions. Conversely, the model’s relatively lower precision (0.81) for large cell carcinoma reflects a higher false-positive rate, likely influenced by the dataset’s class imbalance and the inherent difficulty of distinguishing large cell features, which often lack the distinct histopathologic characteristics of other subtypes [3,9]. Importantly, the model maintained strong recall across all classes, with a maximum of 0.98 for large cell carcinoma and a minimum of 0.85 for adenocarcinoma. These results indicate a low rate of false negatives, an essential feature for lung cancer screening systems, where missed diagnoses can have dire clinical consequences [3]. The uniformly high F1-scores across classes further suggest that the model strikes an effective balance between precision and recall, making it suitable for real-time clinical decision support.

ResNet50V2’s identity mappings and skip connections enable the training of deep models by mitigating vanishing gradient problems, which are particularly critical when classifying subtle differences in CT lung tissue appearance [6]. SE blocks enhance the representational power of CNNs by enabling the model to recalibrate features across channels based on global context, thus prioritizing the most informative representations. This combination has been shown to outperform traditional CNNs in complex image recognition tasks, including tumor segmentation and subtype classification in radiology and pathology [7-10]. Recent studies comparing attention mechanisms, including spatial attention, channel attention, and hybrid approaches, have consistently shown SE blocks to be computationally efficient while offering significant performance gains in medical imaging tasks [8,10]. For example, in MRI-based tumor classification, SE-based models outperformed other attention modules such as the convolutional block attention module (CBAM) and self-attention transformers in both accuracy and convergence stability [8].

From a translational perspective, these results are promising for integration into clinical radiology workflows. The use of deep learning models in radiological interpretation can mitigate some of the most pressing challenges in cancer diagnostics: radiologist shortages, interpretive subjectivity, and diagnostic delay due to high imaging volumes [4,11]. In low-resource settings, where access to experienced thoracic radiologists is limited, such AI systems could offer cost-effective triage support, prioritizing high-risk scans for expert review and facilitating earlier diagnosis and intervention [12]. Moreover, the high discriminative ability of the model supports its potential role in pre-biopsy screening. By offering accurate probabilistic assessments of subtype likelihood, AI tools could inform the choice of biopsy site and modality, potentially reducing the number of unnecessary or nondiagnostic procedures. As lung cancer treatment becomes increasingly personalized based on histologic and molecular subtype, early and accurate subtype classification is essential to guiding targeted therapies and improving patient outcomes [13].

Despite its high performance, the study has several limitations. First, the dataset, though annotated and well-balanced post-weighting, originates from a single publicly available source. It may not encompass the variability introduced by differences in CT scanners, image acquisition protocols, or patient demographics. Therefore, external validation using multi-institutional, real-world datasets is essential before clinical deployment. Second, while SE blocks provided clear benefits in this study, newer attention mechanisms, such as Vision Transformers (ViTs) and hybrid CNN-transformer models, have shown promise in recent literature for capturing long-range dependencies and contextual relationships in medical images [14,15]. Future work could compare these architectures with SE-enhanced ResNets to determine the most effective balance between performance and computational efficiency in diagnostic radiology. Third, although the model achieved high accuracy, it operates as a “black box,” providing limited interpretability. To ensure clinical trust and regulatory approval, future iterations should integrate explainability techniques, such as Grad-CAM or SHAP visualizations, to elucidate which features or regions in the CT images drive predictions [16]. Finally, the current model classifies only four diagnostic categories. Real-world cases often involve mixed pathologies, rare tumor types, or confounding conditions such as infections or fibrosis. Expanding the model to a multi-label or hierarchical classification system could better reflect clinical complexity.

## Conclusions

Overall, SE attention mechanisms on a pre-trained ResNet50V2 architecture for CT-based lung cancer classification demonstrated a test accuracy of 90.16% and an AUC of 0.9815, reflecting strong generalization and discriminative ability across all four diagnostic categories: adenocarcinoma, large cell carcinoma, squamous cell carcinoma, and normal lung tissue. Class-wise performance was particularly notable for normal tissue (AUC = 0.9977, precision = 1.00) and squamous cell carcinoma (AUC = 0.9880), while large cell carcinoma showed high recall (0.98) despite lower precision, highlighting the influence of data set class imbalance and the inherent difficulty of distinguishing large cell carcinoma features. Furthermore, the use of SE blocks enhanced the model's ability to focus on salient imaging features, as reflected in consistently high F1-scores across classes. However, the study is limited by reliance on a single, publicly available dataset without external validation. The absence of multi-center data and diverse imaging sources may introduce sampling bias and limit the model’s robustness across real-world clinical environments. To bridge this gap, future work must validate the model on larger, multi-institutional datasets encompassing varied scanner protocols and demographic backgrounds.

## Appendices

The dataset used containing CT Lung images (n = 1000; classes: adenocarcinoma, large-cell undifferentiated carcinoma, squamous cell, normal CT images) can be found here: https://www.kaggle.com/datasets/mohamedhanyyy/chest-ctscan-images/data.
